# The Evidence before One's Eyes: A Case Report on Schizo-Obsessive Disorder

**DOI:** 10.1155/2012/851785

**Published:** 2012-09-30

**Authors:** Elliott B. Martin

**Affiliations:** Department of Psychiatry, Boston Children's Hospital, 300 Longwood Avenue, Boston, MA 02115, USA

## Abstract

Obsessive compulsive disorder is still considered primarily an anxiety disorder, though historically there has always been a question of whether obsessive-compulsive symptoms may be more properly considered psychotic in nature, the so-called schizo-obsessive disorder or subtype. A case is presented here of a middle-aged man with debilitating obsessive-compulsive symptoms of sudden onset in his late teens. Given the nature of onset and symptomatology, and the failure of prior therapies, the case was approached as a primary psychotic disorder. The neuroleptic-naive patient had remarkable response to low-dose antipsychotic medication, as well as to psychodynamic psychotherapy modeled along the lines of neuroplasticity. The case illustrates the blurred distinctions among anxiety, mood, and psychotic disorders and the improved outcomes when the proper underlying disorder is addressed.

## 1. Introduction

Obsessive compulsive disorder (OCD), at least in the waning days of DSM-IV, is still considered phenomenologically an anxiety disorder. The connection between OCD and psychotic disorders, however, has been noted for well over a century [1], and the psychiatrically colloquial term “schizo-obsessive” has been in the literature, if not in the diagnostic subtypes, for many years [2]. The diagnostic dilemmas produced by the DSM's allowance of “with poor insight” as a specifier have been previously noted [3], especially with regard to differentiating among delusions, obsessions, and overvalued ideas [4]. There is discrepancy between earlier studies of the prevalence of OCD and schizophrenia comorbidity and more recent studies, with researchers from the first half of the 20th century putting the rate at 1–3.5% [5] and latter day researchers, after controlling for medication-induced OCD, putting the rate at about 14% [6, 7]. Of greater interest, however, are the earlier theories as to obsessive-compulsive symptoms being an effective defensive mechanism against psychotic symptoms, at times even allowing for remission of schizophrenia altogether [5, 8–10]. This theory has obviously been disregarded in the wake of neurotransmitter theories and the investment in the fMRI [11]. However, a case is presented here of severe and debilitating OCD with a late adolescent onset so striking that the working hypothesis in treatment, as to etiology, was indeed that the patient had constructed a massive obsessional-compulsive architecture in order to ward off (successfully) what seemed, from history, to be repeated psychotic breaks as a young man in his teens and early 20s.

## 2. Case Presentation

On initial outpatient presentation to our community mental health center patient was a 41-year old, college-educated single male, with a twenty-four-year history of severe OCD and depressive symptoms. Other than his former outpatient psychiatrist continuing his monthly prescription for Zoloft he had had no consistent psychiatric followup in the community for over ten years before coming to us. The patient's chief initial complaint was worsening depression in the context of the recent termination of a romantic relationship—the 19-year-old escort he had been seeing on a monthly basis for six months had since refused to take his calls, to respond to his texts, or to see him anymore. The patient had been attending a monthly OCD support group at our center for several months before this and learned of the possibility of individual psychiatric care via Internet search. 

The patient had no history of inpatient hospitalization, no history of suicide attempt or other violence. Suffering from obsessive-compulsive symptoms since age 17, he has been receiving state and federal assistance for disability secondary to the disorder since age 28. The patient breaks down his current OCD behavior into four categories: (1) a poking sensation in the jaw as if with a hanger, (2) skin and hair, especially his beard, so obsessed with thoughts of trimming his beard that he often appears thought-blocked during conversation, (3) preoccupations w/scratches on glass surfaces or other surfaces with a sheen, and (4) obsessions with women and ideas of physical perfection.

The patient also has significant hoarding behaviors. Unable to live in his state-subsidized apartment any longer secondary to clutter, he splits his time between his divorced parents' homes. Currently it takes the patient several hours to groom daily; he showers two times per week secondary to the length of time required, moves his bowels every three days for the same reason. He eats one meal a day, never wears a coat, and never exercises. He no longer brushes his teeth due to the time it takes—has monthly cleanings. He no longer masturbates secondary to the lengthy cleaning afterward. He does see an escort about once per month. Though he denies ever having experienced sexual side effects from Zoloft, he takes a med holiday four days before planned sexual activity to maximize the experience. Extremely well read on OCD, the patient often contributes to newsletters; he quotes studies, papers, and authorities frequently. 

The patient's depressive symptoms vary with the intensity of particular OCD symptoms, overall well managed on Zoloft. The suggestion of atypical antipsychotics as medication augmentation had previously been met with pleasant but firm resistance. Pt has rituals around medication taking, and this has limited pharmacotherapeutic interventions.

The patient was first diagnosed with OCD at age 17. He reports the onset was sudden, that he was sitting in his high school gym during a film session for football practice when his attention was drawn to a wire coat hanger hanging on the back of a door. He became suddenly and powerfully obsessed with the thought of the hanger uncoiled and poking him in the jaw from the inside out. So vivid was this thought that he reports actually feeling the pain of this. From that point on his obsessions grew and flourished with accompanying, evolving compulsions. The one constant in his history is this poking sensation that has never remitted. The patient, who prides himself on an outstanding memory, has much difficulty recalling the events of his history between ages 17 and 18. He does recall, however, worsening symptoms during his freshman year of college at a top-tier university; he saw therapists and behavioral specialists at two of the top such facilities in the world during his undergraduate years and had multiple med trials at that time, all with modest success. 

The patient also reports another especially noteworthy episode during his junior year of college in which he initiated electrolysis treatment in order to manage his facial grooming better. He adamantly denies this was part of any exposure therapy. He reports that during the first minute of his very first treatment he panicked at the sight of redness on his face, a temporary effect of which he had been well warned. He ran from the facility, hyperventilated in his car, and reports that this incident opened the floodgates of anxiety with which he has been struggling since. He has not shaved since.

The patient next saw a psychoanalyst for several years after college, who also placed him on the Zoloft he still currently takes. He intermittently worked in professional capacities in Boston and New York, returning to his parents' home in between jobs, seeing different clinicians in each location. He never sustained employment for longer than a year and never was promoted beyond entry level. He did become involved in romantic relationships as a younger man, none lasting longer than several months. He has seen exclusively escorts since 2005. After the patient was awarded public assistance in 1999, he was unable to afford further treatment, and as he put it, “I was free to explore my obsessions and compulsions to their logical conclusions.” The intensity and length of rituals increased substantially. The only therapeutic interaction at this time was his attending various support groups in his region, groups at which he enjoyed taking on a senior role to those with “OCD-light.”

The patient has no history of psychiatric hospitalizations. He has no history of suicidality or homicidality, though he frequently talks philosophically/existentially of suicide. Ironically, he points to his atheism as his main protective factor against suicide, stating that he knows this is all he has, and so he would never kill himself. He has no significant substance use history. There is a diffuse history of depression and anxiety on both sides of the patient's family, with patient's mother and maternal grandmother receiving medication treatment.

The patient's medical history is significant for a concussion received while playing football a week before onset of OCD. There was no loss of consciousness or hospitalization, and patient reports no adverse effects otherwise. Otherwise there is no other significant medical history, and patient was taking no other medications.

Developmentally, the patient and his mother deny any pre or perinatal issues. Patient reports toilet training was problematic, though mother denies any issue. There is no history of physical or sexual abuse, though patient states that his father had “a terrible temper,” and was “emotionally unequipped to deal with work and a family.” At one time stated he “hated” his father as a child. His only sibling is a younger brother, “the picture of normalcy;” he owns his own business, is married, with two young children. Pt reports a positive relationship with his niece, nephew, and sister-in-law, as well as supportive relationships with his mother and brother as a child. His parents both are educators, and he attended public school till 8th grade. He then attended an exclusive private school for high school, repeating his freshman year secondary to “poor adjustment”—mother states the patient had difficulty with the transition from public to private school. Patient admits to being keenly aware of the financial discrepancies around him at that school. He was a varsity athlete, with good academic performance after freshman year, no truancy. Despite being in treatment, patient graduated college in four years with an education degree. 

The patient, unable to live in his apartment secondary to hoarding, currently lives alternate weeks between his now divorced parents' homes. He still maintains several close friendships from high school and college and entertains aspirations for a literary career. He remains politically engaged, reading up on current issues and maintaining strong opinions, and also remains engaged in the latest literature on OCD.

## 3. Discussion

Pt was initially engaging, obviously intelligent, and appeared at first to be insightful and eager for treatment. Treatment was initially difficult, however, in that the patient viewed himself as “trapped in this OCD body,” very clearly resigned to his fate. He very much intellectualized his predicament, describing his obsessions, compulsions, and anxieties matter-of-factly, and at great length. He made it clear from the outset that he was not interested in behavioral therapies: “No offense, Doc, but I've been to the best in the world, and they haven't been able to help me.” A psychodynamic approach was thus employed.

As therapy progressed it became clear that the pt was more invested in his OCD than in trying to overcome it. He pleasantly but firmly resisted all attempts at psychopharmacologic treatment. He firmly resisted all attempts at behavioral therapies. The narcissistic nature of insight-oriented therapy obviously appealed to him. But the patient did not display any significant signs of narcissistic personality disorder. He has always been quite cordial and mannerly. He listens. He is grateful and has never been demanding. He is, however, susceptible to narcissistic injury, and it became evident during the termination phase with this therapist that a fair amount of nonspecific anger simmered below the surface.

In seeking treatment alternatives what was most striking in the patient's history was the sudden dramatic onset of the disease in the locker room at age 17, the hazy memories thereafter, and the reinforcement of it two years later with a second powerfully bizarre episode during electrolysis. These are the two episodes that the patient considers most noteworthy in his history, both with continued repercussions—the poking sensation remains, and the patient still will not shave. Especially the poking sensation has been approached as more of a somatic delusion, and working from there, the approach has been that of considering this a primary psychotic disorder, that indeed the patient had successfully warded off a psychotic break at age 17 and again at age 19 by constructing an elaborate system of obsessions and compulsions. This was discussed candidly with the patient, who always requested openness from the therapist. He remained thoughtful as to the possibility. Evidence in favor of this approach was that the patient had a remarkable response to Risperdal augmentation, even at low doses. It was a struggle to get him to agree finally to augmentation strategy, and at a dose of 0.5 mg at night pt reported dramatic improvement in mood, if not so much his OCD. He began to request dose increases himself, though such has been hampered by his rituals around med taking. Currently up to 2 mg at night there has been some subjective improvement in ritualistic behaviors as well as sustained improvement in mood. 

Psychotherapeutically, the approach has been to follow more the work of Jeffrey Schwartz on neuroplasticity in OCD [12]. This approach is generally the opposite of traditional behavioral therapies in that essentially the OCD is ignored completely in session in efforts to reconstitute neural pathways away from such. Dr. Schwartz typically employs mindfulness in his sessions, but in this case a more conventional psychodynamic course was chosen. This had the notable effect that the patient, after several weeks, hardly performed any rituals in session. 

The patient was treated weekly for a year with such modalities before this writer, a trainee, had to move on to fellowship at another facility. Improvement was measured psychosocially in that patient over the course of the year reported he was able to watch sports again (which he had not been able to do since his breakup with the escort), that he was able to see escorts again (measuring success in odd ways) and that his relationship with his father had improved substantially. Further evidence for a primary psychotic disorder was made apparent during the termination phase, in which the patient variously, and uncharacteristically, showed for session either in an absolute fury or morbidly depressed. The depth of underlying emotion was thereby evidenced as profound, so much so that the patient, again very much out of character, at one time called the local suicide hotline and at another went through the screening process to have himself admitted to a local inpatient research study. These anxieties were successfully addressed with a family meeting involving the patient, his parents, this clinician, and the incoming clinician. The option of psychosurgery was openly discussed with the patient and his family, and the process of reeducating the patient how to interpret more negative thoughts, such as those of irritation and anger, in a nonruminative or nonobsessional way has been initiated. The patient has confessed that if his OCD were to just go away, “I don't know what I'd do.”

By way of conclusion, this case illustrates the complexity of overlap among mood, anxiety, and psychotic disorders. But it also illustrates that a successful targeting of the main underlying problem can have dramatic effects, even after years of unsuccessful treatment. This case is also somewhat of a small vindication of the early theorists on schizophrenia, those who suggested that obsessive-compulsive symptoms may in fact be protective against psychosis [5, 8–10]. And briefly to address the suggestion of PANDAS, a search has not revealed a case in the teenage years.
